# Stochastic Mechanical Response and Failure Mode Transition of Corroded Buried Pipelines Subjected to Reverse Faulting

**DOI:** 10.3390/ma19051033

**Published:** 2026-03-08

**Authors:** Tianchong Li, Kaihua Yu, Yachao Hu, Ruobing Wu, Yuchao Yang, Feng Liu

**Affiliations:** 1 College of Civil Engineering and Architecture, Shandong University of Science and Technology, Qingdao 266590, China; 2 School of Civil Engineering, Shandong Jianzhu University, Jinan 250101, China; 3 School of Civil Engineering, Southeast University, Nanjing 211189, China

**Keywords:** buried pressurized pipeline, spatial corrosion heterogeneity, conditional diffusion model, reverse faulting, failure mode transition, probabilistic safety assessment

## Abstract

Buried oil and gas pipelines, the critical arteries of global energy infrastructure, are increasingly vulnerable to severe geological hazards such as reverse faulting, yet their structural integrity is often pre-compromised by stochastic corrosion damage accumulated during service. However, quantifying the coupled impact of spatial corrosion heterogeneity and large ground deformation remains a formidable challenge due to the complex nonlinearities involved in soil–structure interactions and wall thinning. This study establishes a probabilistic assessment framework integrating random field theory, nonlinear finite element analysis, and a generative conditional diffusion model to characterize realistic 2D non-Gaussian corrosion morphologies. The numerical results reveal a significant geometric stiffening effect induced by internal pressure, where moderate operating levels effectively suppress cross-sectional distortion by counteracting the Brazier effect. Consequently, this mechanism facilitates a fundamental transition in failure modes from localized tensile rupture to ductile buckling, significantly extending the critical fault displacement threshold. Furthermore, probabilistic fragility analysis demonstrates that the spatial dispersion of pitting, rather than just average wall thinning, governs the initiation of premature failure. Mechanistic analysis indicates that high internal pressure, while providing pneumatic support, exacerbates tensile strain localization at corrosion pits, leading to a heightened probability of premature rupture under minor fault deformations, a critical hazard that traditional deterministic models significantly underestimate. These findings provide a quantitative theoretical foundation for the reliability-based design and maintenance of energy lifelines traversing active tectonic zones.

## 1. Introduction

Buried pipelines act as critical arteries for energy transmission—transporting oil and natural gas—and constitute an essential component of modern lifeline infrastructure. However, these long-distance cross-regional pipelines inevitably traverse seismically active fault zones. Among various geological hazards, fault displacement poses the most severe threat to buried pipelines [1]. Reverse faulting subjects the pipeline crossing the fault to intense axial compression and bending loads. This loading condition makes the structure highly susceptible to local buckling, severe wrinkling, and potentially rupture, leading to catastrophic energy leakage and secondary disasters [2].

Concurrently, during long-term service, pipe walls are unavoidably subject to corrosion due to electrochemical processes or microbial erosion in the soil environment. Corrosion not only reduces wall thickness but, more importantly, induces a reduction that is significantly random and non-uniform in its spatial distribution. This spatial heterogeneity of wall thickness alters the strain localization patterns during loading, thereby significantly impairing the pipeline’s capacity to withstand large fault deformations and potentially changing the ultimate failure mode [3,4]. Furthermore, transmission pipelines typically operate under internal pressure. The presence of internal pressure exerts a complex dual influence on the mechanical response: on one hand, the hoop stress generated by internal pressure increases the pipeline’s buckling resistance and mitigates cross-sectional ovalization; on the other hand, excessive internal pressure exacerbates tensile stress in the pipe wall. Under reverse faulting, this may cause the failure mode to shift from a buckling-dominated mechanism to a rupture-dominated one [5]. Therefore, safety assessments of pipelines crossing faults must comprehensively consider the coupled effects of reverse faulting movement, non-uniform corrosion, and internal pressure.

Despite extensive academic research on the mechanical response of buried pipelines, existing findings remain significantly limited when addressing multi-factor coupling problems involving corrosion and internal pressure. These limitations are primarily evident in three aspects.

First, research on fault-crossing buried pipelines has predominantly focused on pristine pipes or those with idealized defects. For instance, Zhang et al. [6] established a refined finite element model using ABAQUS to systematically study the effect of different fault crossing angles on the strain distribution of X80 steel pipes; however, their study was limited to defect-free pipelines. Similarly, while recent work by Han et al. [7] explored pipeline failure modes under reverse faulting—noting that reverse faults are more likely to induce local buckling than strike-slip faults—the study did not account for corrosion degradation effects following long-term service. Other scholars, such as Banushiet al. [8], considered fault displacement responses in buried pipelines using numerical simulations, yet their models relied on deterministic geometric parameters and neglected corrosion damage.

Second, existing studies on corroded pipelines often rely on simplified uniform corrosion assumptions, failing to capture the spatial variability of realistic corrosion morphologies. Bao [9] highlighted that natural corrosion is highly random, and uniform thickness reduction models typically underestimate failure risks. To address this, Chen et al. [10] used random field theory to simulate the spatiotemporal evolution of corrosion, confirming the importance of spatial correlation. However, current morphology modeling methods struggle with precise roughness control. Traditional random field models rely on limited statistical indicators (e.g., correlation length, variance, or fractal dimension), which only allow for indirect adjustments to surface roughness. Approaches using Karhunen–Loève (KL) expansion [11,12], wave trains, or fractal reconstruction [13] focus primarily on macroscopic morphology and cannot explicitly control common engineering roughness parameters. While random field perturbation methods can adjust pit depth or amplitude, they still lack a comprehensive roughness control system [14]. To overcome these limitations, this study proposes a deep generative approach. A conditional diffusion model is employed to learn the complex nonlinear mapping from a 12-dimensional roughness parameter vector to the Power Spectral Density (PSD), thereby enabling specific engineering indices to serve as direct inputs. Furthermore, while previous research on random corrosion fields (e.g., Zhao [15], Khakzad [16]) primarily focuses on burst failure under internal pressure or simple bending, few studies have applied complex 2D corrosion mapping to analyze large-deformation buckling induced by reverse faulting.

Finally, there is a lack of a systematic assessment framework that integrates internal pressure coupling effects with probabilistic uncertainty. Internal pressure has a decisive influence on the failure modes of fault-crossing pipelines. Bi et al. [17] demonstrated that while high internal pressure suppresses wrinkling deformation, it accelerates the onset of tensile rupture; this competitive mechanism becomes increasingly complex in the presence of corrosion. Although Liu et al. [18] proposed a probabilistic assessment framework for the seismic performance of buried pipelines, emphasizing the importance of multi-source uncertainties, a Monte Carlo simulation study that simultaneously integrates spatially correlated non-Gaussian corrosion random fields, internal pressure, and nonlinear soil-pipe interaction is still missing. Most existing works (e.g., Teng et al. [19]) continue to rely on deterministic safety factor methods, which struggle to quantify the contribution of each random variable to the ultimate failure probability of the pipeline.

To address these deficiencies, this paper establishes a comprehensive assessment framework for fault-crossing pipelines that fuses random field theory with nonlinear finite element analysis. The remainder of this study is structured as follows: Section 2 details the probabilistic framework for corrosion modeling, where random harmonic functions are utilized to generate a wall thickness random field with spatial correlation, accurately characterizing realistic corrosion morphology. Section 3 describes the construction of a high-fidelity fluid–structure coupling finite element model, where non-uniform corrosion data are mapped onto shell elements and combined with fluid cavity techniques to elucidate the joint driving mechanisms of corrosion and internal pressure. Section 4 provides a validation of the numerical methodology against experimental benchmarks. Section 5 presents a systematic investigation into the distribution laws of fracture strain and failure modes through large-scale Monte Carlo simulations, quantifying the structural response under multiple operating conditions. Finally, Section 6 summarizes the key findings and provides a theoretical basis for the reliability design and maintenance of pipelines crossing faults.

## 2. Random Corrosion Field Modeling

### 2.1. Corrosion Testing and Surface Characterization

To accurately replicate the stochastic nature of realistic corrosion morphologies in numerical simulations, this study utilizes morphology data obtained from electrochemical corrosion tests. The data were derived from previous work involving controlled accelerated electrochemical corrosion tests performed on 24 pipe segment specimens, which specifically simulated four distinct service environments: seawater, clay, sandy soil, and gravel [20]. By applying a constant direct current, the experiments simulated the long-term electrochemical degradation process of buried pipelines in various media, aiming to capture the spatial distribution characteristics of corrosion depth under realistic environmental conditions.

The surface morphology of the corroded pipe walls was acquired using a Handyscan handheld self-positioning 3D laser scanning system manufactured by Creaform Inc., Lévis, QC, Canada. However, the raw point cloud data contained initial macroscopic geometric deviations from the manufacturing process, such as ovality and uneven wall thickness; direct usage of this data would interfere with the characterization of corrosion stochasticity. Therefore, this study employed Gaussian filtering techniques to perform frequency domain separation on the unwrapped surface elevation data. This process eliminated low-frequency geometric deviations, thereby extracting a “relative thickness” field containing exclusively corrosion information.

**Figure 1:**
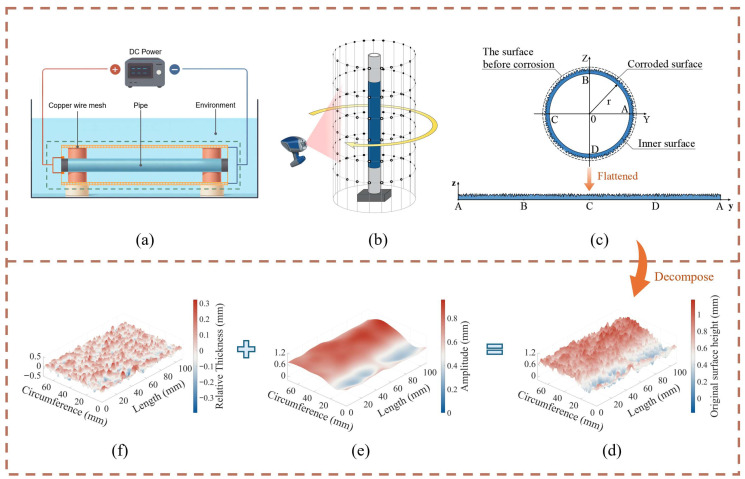
Framework of corrosion data acquisition: from electrochemical acceleration to signal separation via Gaussian filtering [20]. (**a**) Schematic of the electrochemical corrosion setup; (**b**) The outer surface of the corroded pipe was scanned 360° using a three-dimensional laser scanner; (**c**) Processing point cloud data of the corroded pipe and flattening the pipe surface; (**d**) Unwrapped original topography of the corroded pipe; (**e**) Initial geometric shape extracted from the original topography after Gaussian filtering; (**f**) Corrosion topography obtained after removal of the geometric shape.

Subsequently, based on the calculation methods provided by ISO 25178 [21], 12 key surface roughness parameters were extracted from each filtered sample. These parameters quantify the statistical characteristics of the corrosion texture, providing physical constraints for the subsequent conditional generation model. Figure 1 illustrates the data acquisition workflow, spanning from the electrochemical experiments to signal separation.

### 2.2. Two-Stage Generative Simulation Method

Building upon the aforementioned high-fidelity experimental data, this section develops a two-stage generation method integrating a conditional diffusion model with Second-order Harmonic Functions (SHF-II). By leveraging a deep generative model to capture the complex correlation between roughness parameters and frequency domain characteristics, the random corrosion fields required for this study are simulated.

To construct a robust training dataset, a sliding window sampling method was employed to partition the raw large-format scan data, yielding a total of 16,872 local corrosion samples and their corresponding roughness condition vectors. Utilizing the Guided Diffusion framework proposed by OpenAI [22], a conditional diffusion model based on the U-Net architecture was trained to establish a nonlinear mapping from roughness statistical parameters to the Power Spectral Density (PSD).

**Figure 2:**
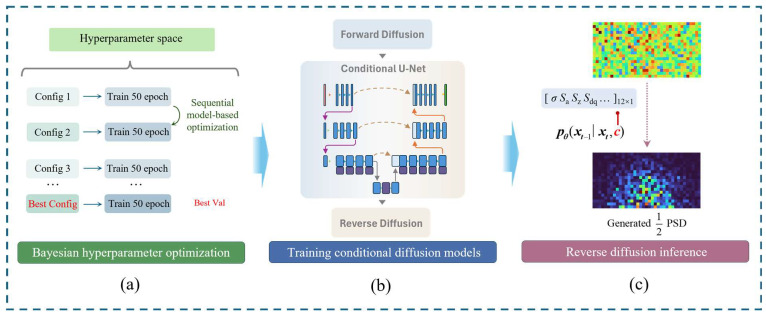
Schematic diagram of the proposed conditional diffusion model framework. (**a**) Bayesian hyperparameter optimization, which employs sequential model-based optimization to identify the best configuration through iterative 50-epoch training trials. (**b**) Training of the conditional diffusion models, illustrating the forward and reverse diffusion processes implemented via Conditional U-Net architecture. (**c**) Reverse diffusion inference for PSD generation, where the model reconstructs the 1/2 PSD from noise, guided by the specific environmental condition vector.

As illustrated in Figure 2, considering the centrosymmetry of the PSD matrix, only the semi-region (one-half) was extracted as the target tensor during training. This strategy significantly reduced computational overhead while ensuring information integrity. Model training was driven by minimizing the Mean Squared Error (MSE) of the noise prediction; the loss function, $L_{simple}$, is defined as:

(1) $$L_{\mathrm{simple}}=E_{t,x_{0},\epsilon }\left[||\epsilon -\epsilon_{\theta }(x_{t},t,c)|{|}^{2}\right]$$

where t denotes the diffusion time step; $x_{0}$ represents the ground-truth PSD data; and ϵ~N(0,I) is random noise sampled from a standard normal distribution. Furthermore, $x_{t}$ corresponds to the noisy data at step *t*, while $\epsilon_{\theta }(x_{t},t,c)$ signifies the noise component predicted by the network, conditioned on the roughness parameter *c*. This loss function ensures that the model accurately learns the conditional probability density of the frequency domain energy distribution across varying corrosion environments.

As illustrated in Figure 2a, to achieve optimal model performance, a Bayesian hyperparameter optimization strategy [23] was implemented. In this strategy, we performed a search within a multidimensional parameter space to identify the globally optimal combination of hyperparameters—including learning rate, batch size, and diffusion steps—thereby ensuring the model’s generalization capability across diverse environmental data.

**Figure 3:**
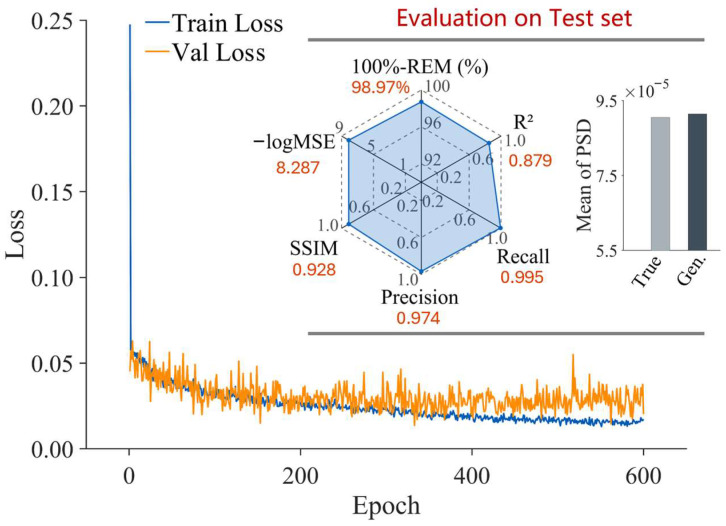
Training and validation loss curves of the conditional diffusion model, together with evaluation results on the test sets.

As illustrated in Figure 3, both the training and validation loss curves exhibit a monotonic decrease and converge to a stable state. The close alignment between the two curves indicates that the model has effectively mitigated the risk of overfitting. A comprehensive quantitative assessment was performed on the test set using multiple metrics, including Relative Error Mean (REM), Mean Squared Error (MSE), Coefficient of Determination ($R^{2}$), and Structural Similarity Index Measure (SSIM). The evaluation demonstrates that the model achieves superior performance across all investigated conditions, specifically, the $R^{2}$ values consistently exceed 0.85, while the SSIM approaches 1. These results substantiate the model’s capability to accurately capture both the amplitude distribution and the spatial structure of the PSD.

In contrast to traditional analytical spectra and established stochastic surface modeling techniques, the PSD generated by the diffusion model offers a distinct advantage in explicitly controlling specific morphological parameters. Traditional methods often require iterative adjustments to match target roughness statistics or assume Gaussian distributions that may not capture the complex non-Gaussian nature of real corrosion. In this study, the AI-driven approach allows for the direct generation of high-fidelity corrosion surfaces conditioned on predefined roughness parameters (such as STD). This capability is crucial for systematically isolating the effect of spatial dispersion on mechanical response while keeping the average remaining thickness constant. Furthermore, trained on a diverse dataset of 16,872 experimental samples, the model ensures high fidelity across a wide range of conditions, capable of simulating both low-STD surfaces (representing uniform corrosion) and high-STD surfaces (representing pitting), with the flexibility to extend to more complex roughness parameters in future analyses. Subsequently, the generated PSD serves as the target power spectrum and is input into the SHF-II algorithm to synthesize spatial random fields [24,25]. As a spectral representation technique, SHF-II transforms frequency domain information into a spatial domain height field by superposing a series of trigonometric function terms characterized by random phases and amplitudes.

The reconstruction formula for the corrosion depth, $h(x_{j},y_{j})$, at the spatial coordinate $\left(x_{j},y_{j}\right)$ is expressed as:

(2) $$h(x_{j},y_{j})=2\sqrt{2}\sum_{u=0}^{M-1}\sum_{v=0}^{N-1}A_{u,v}\left[\cos(k_{x,u}x_{j}+k_{y,v}y_{j}+\phi_{1,u,v})+\cos(k_{x,u}x_{j}-k_{y,v}y_{j}+\phi_{2,u,v})\right]$$

where M and N represent the number of cut-off terms in the frequency space; $A_{u,v}$ denotes the amplitude of the frequency component determined by the generated PSD; $k_{x,u}$ and $k_{y,v}$ are the corresponding discrete wavenumbers; and $\phi_{1,u,v}$ and $\phi_{2,u,v}$ are independent random phase angles following a uniform distribution over the interval [0,2π).

This mathematical formulation not only ensures that the generated samples strictly adhere to the spectral characteristics of the target PSD but also endows the model with high spatial flexibility, enabling the generation of corroded pipe surfaces with arbitrary resolutions and dimensions to accommodate finite element meshing requirements. Figure 2 illustrates the complete architecture of this generation framework.

**Figure 4:**
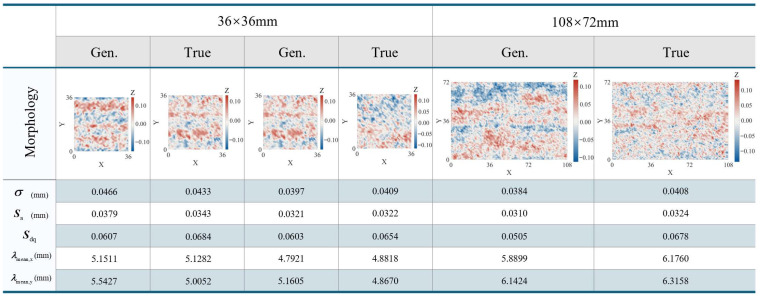
Morphology and roughness comparison between typical simulated and real samples for small-scale 36 × 36 mm and large-scale 108 × 72 mm surfaces.

Figure 4 presents a comparison of the morphology and roughness between a typical small-scale (36 × 36 mm) random field and a large-scale (108 × 72 mm) corrosion surface. The results indicate that, at the small scale, the simulated surface pit morphology, texture orientation, and fluctuation amplitude are highly consistent with the measured samples. Furthermore, roughness parameters such as $S_{a}$, $S_{z}$, σ, and $S_{\mathrm{dq}}$ closely match those of the real specimens.

Regarding the large scale, the SHF-II method successfully generates surfaces of arbitrary dimensions by adjusting grid dimensions and sampling intervals, while preserving the statistical structure defined by the PSD. The simulated long-wave undulation patterns align well with the global fluctuations of the real corrosion surface, effectively demonstrating the method’s superior scalability.

To accommodate simulation requirements across varying scales, one need only adjust these parameters while maintaining the input PSD constant. This approach enables the generation of corrosion surfaces that possess identical statistical properties yet differ in spatial dimensions, thereby significantly facilitating multi-scale corrosion modeling and practical engineering applications.

## 3. High-Fidelity Numerical Framework: Pipe–Soil–Fluid Interaction and Stochastic Mapping

This study focuses on a representative high-strength steel buried gas pipeline traversing a fracture zone associated with a reverse fault. The engineering problem is idealized as a pipe–soil interaction system subjected to Permanent Ground Deformation (PGD). The prototype pipeline utilizes API 5L X65 grade steel with an outer diameter (D) of 101.6 mm and a nominal wall thickness ($t_{nom}$) of 3.6 mm; these specifications are characteristic of modern high-pressure natural gas transmission trunk lines. This specific diameter-to-thickness ratio aligns with actual engineering applications, thereby ensuring the cross-scale applicability of the findings. Mechanically, this cross-scale equivalence is justified because the localized buckling and shell instability behaviors of buried pipelines are primarily governed by this dimensionless parameter rather than their absolute physical dimensions. The pipeline is buried within a homogeneous soil stratum with a burial depth to the pipe crown set at 0.3 and 1.2 m. To simulate the reverse faulting scenario, a ‘split-box’ geometric model is employed. The fault dip angle (β) is defined as $60^{{}^{\circ}}$ with respect to the horizontal plane. The fault displacement ($\Delta_{f}$) is imposed via the movement of the footwall relative to a stationary hanging wall, which resolves into significant axial compression and vertical shear components along the pipeline axis.

### 3.1. Finite Element Model

To investigate the mechanical response of buried pipelines subjected to soil–structure interaction, a three-dimensional finite element model was developed using the ABAQUS 2019 commercial software package. The numerical model comprises two primary components: the pipeline and the surrounding soil assembly. A quasi-static analysis was performed using an explicit dynamic integration scheme with geometric nonlinearity enabled. This approach was adopted to effectively handle the severe nonlinearities associated with large deformations and complex multi-contact interfaces.

#### 3.1.1. Pipe Modeling

**Figure 5:**
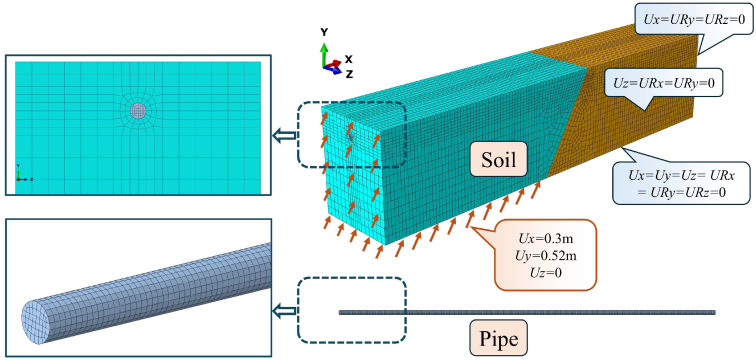
High-fidelity finite element architecture of the buried pipeline–soil system.

The pipeline was discretized using three-dimensional four-node reduced-integration shell elements (i.e., S4R). For the corroded scenarios, the shell section thickness of each individual element was determined via the random corrosion field mapping method, whereas a constant wall thickness of 3.6 mm was assigned for the pristine (non-corroded) condition. Nine integration points were employed through the shell thickness to ensure the accurate integration of strain gradients associated with bending and local buckling. Based on a mesh sensitivity analysis, an average characteristic element length of 15 mm was selected for the pipeline. The schematic of the finite element model is presented in Figure 5.

**Figure 6:**
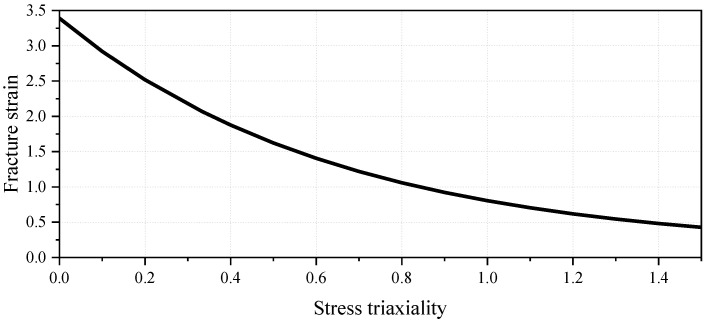
Fracture Locus of API 5L X65.

The pipeline was modeled using API 5L X65 grade steel. An elastoplastic constitutive model incorporating an isotropic hardening rule was adopted to characterize the material behavior. The specific material parameters [26] are listed in Table 1. The material fracture and failure behavior was simulated using the built-in ductile damage model in Abaqus. Since the equivalent plastic strain at fracture for X65 pipeline steel is highly dependent on the stress state, the fracture strain was defined as an exponential function of stress triaxiality. The following fracture criterion was adopted [27]:

$$\varepsilon_{f}=3.29\exp\left(-1.54\eta \right)+0.1$$

where *η* denotes the stress triaxiality, defined as the ratio of the hydrostatic pressure to the equivalent stress. The fracture locus is shown in Figure 6.

To accurately account for inherent deformations arising from manufacturing, installation, and service history in numerical simulation, initial geometric imperfections were introduced into the pipeline model. Prior to the nonlinear analysis, an eigenvalue buckling analysis was performed on the pipeline subjected to uniform hydrostatic pressure to extract representative global and local buckling modes. Subsequently, the selected modes were scaled to a specified amplitude, and the resulting displacement field was superimposed onto the perfect geometry to define the initial configuration for the subsequent nonlinear analysis. This methodology is widely adopted in research concerning the buckling behavior of buried pipelines [28,29]. In this study, the first-order global buckling mode and the mode dominated by local buckling were selected for superposition, with the imperfection amplitude set to L/1000.

#### 3.1.2. Soil Modeling and Pipe–Soil Interaction

The surrounding soil was discretized using three-dimensional eight-node reduced-integration solid elements (i.e., C3D8R), as illustrated in Figure 5. Mesh refinement was applied to the soil region in direct contact with the pipeline, and the average characteristic element lengths for the refined and non-refined zones were set to 65 mm and 100 mm, respectively. In the model assembly, the two soil blocks (representing the hanging wall and footwall) were defined as independent parts. This configuration was adopted to facilitate relative sliding and separation (gap opening) behaviors at the fault interface during the faulting process.

The mechanical behavior of the soil was characterized by the elastoplastic Mohr–Coulomb constitutive model. The corresponding material parameters [28] are listed in Table 2.

Contact relationships were established for both the steel pipe–soil interface and the soil–soil interface. For the normal behavior, a “hard contact” formulation was employed, allowing for separation and subsequent re-contact between surfaces. The tangential behavior was characterized using the Coulomb friction model, with a friction coefficient of 0.41 [26].

#### 3.1.3. Boundary Conditions and Loads

Figure 5 also illustrates the boundary conditions applied to the finite element model. The soil domain was partitioned into a stationary footwall block and a moving hanging wall block, intersected by a fault plane with a dip angle of 60°. For the stationary footwall block, all degrees of freedom (DOFs) on the bottom surface were constrained to simulate a rigid bedrock boundary. On the lateral boundaries, constraints were applied to the translational DOF in the z-direction (*U*_z_ = 0) and rotational DOFs about the x- and y-axes (*UR*_x_ = *UR*_y_ = 0) to represent lateral soil confinement. Similarly, the longitudinal end faces were constrained against translation in the x-direction (*U*_x_ = 0) and rotation about the y- and z-axes (*UR*_y_ = *UR*_z_ = 0) to simulate the continuity of the far-field soil. For the moving hanging wall block, displacement boundary conditions were applied to the bottom, lateral, and end surfaces. These displacements were imposed using a linear amplitude function to drive the soil block along the vector of the fault plane, thereby simulating the reverse faulting movement.

It should be noted that, while variables such as the fault crossing angle and soil constitutive properties inherently possess uncertainties, they are treated deterministically in this study to isolate the probabilistic structural impact of corrosion morphology, internal pressure, and burial depth. The adopted deterministic values for the fault crossing angle and soil constitutive parameters are reasonable and consistent with classical quasi-static pipeline–fault interaction assessments [30,31].

### 3.2. Mapping Random Corrosion Field to FE Model

To convert the generated two-dimensional continuous random fields into discrete geometric attributes compatible with the finite element model, this study developed an automated mapping algorithm. This algorithm serves as an interface between the stochastic process and structural analysis, facilitating the precise projection of pixel data from the random field onto the thickness attributes of the finite element shell elements.

The primary task of the mapping program is to construct the geometric topology of the pipeline. Based on the macroscopic dimensions of the pipeline (diameter *D* and length *L*), the algorithm generates a regular structured quadrilateral mesh on a two-dimensional unfolded plane. A mesh size of 15 × 15 mm was selected; this resolution balances the need to capture local pitting corrosion details with the effective control of computational degrees of freedom. Subsequently, a cylindrical coordinate transformation wraps the node coordinates (x,y) from the 2D plane into 3D Euclidean space (X,Y,Z), forming the final cylindrical shell configuration by

(3) $$\left\{\begin{array}{l} X=x\\ Y=(D/2)\cos(2y/D)\\ Z=(D/2)\sin(2y/D) \end{array}\right.$$

Within this geometric framework, the spatial discretization of corrosion depth adopts the “Element-Centroid Sampling” method. The algorithm iterates through each generated finite element, calculates the corresponding coordinates of its geometric centroid within the 2D random field based on topological connectivity, and extracts the local corrosion depth value, $d_{corr}$, at that location using bilinear interpolation. The effective remaining wall thickness, $t_{elem}$, for each element is defined as the difference between the nominal pipeline wall thickness, $t_{nom}$, and the local corrosion depth:

(4) $$t_{elem}^{\left(i\right)}=\max\left(t_{nom}-d_{corr}(x_{c}^{\left(i\right)},y_{c}^{\left(i\right)}),\;t_{min}\right)$$

where $\left(x_{c}^{\left(i\right)},y_{c}^{\left(i\right)}\right)$ denotes the centroid coordinates of the i-th element. To prevent negative thickness values or numerical singularities caused by excessive corrosion, a truncation threshold $t_{min}$ (set to 0.1$t_{nom}$) was introduced to ensure that all elements remain physically valid.

**Figure 7:**
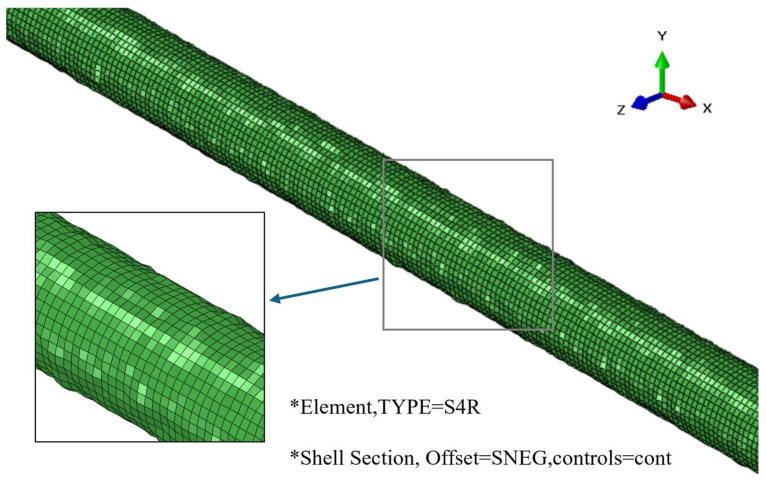
Finite element model of the pipeline with spatially varying shell thickness mapped from the random corrosion field. The asterisks (*) in the figure are used to denote key modeling definitions: *Element specifies the element type (S4R shell element), and *Shell Section defines the shell section properties, including offset and control parameters.

Finally, this algorithm bypasses the tedious process of solid geometry modeling. Instead, it directly synthesizes the input file (.inp) required by the ABAQUS solver. By defining node sets and element connectivity, tens of thousands of calculated discrete thickness values are encapsulated within the *SHELL SECTION keyword. The resulting pipeline finite element model, characterized by a non-uniform element thickness distribution, is illustrated in Figure 7.

This modeling strategy, based on discrete thickness fields, effectively circumvents the risk of element distortion commonly associated with the solid meshing of complex defects. Furthermore, it significantly reduces the computational overhead required for stiffness matrix assembly, thereby establishing an efficient and robust numerical foundation for the subsequent large-scale Monte Carlo simulations.

### 3.3. Modeling of Internal Pressure

**Figure 8:**
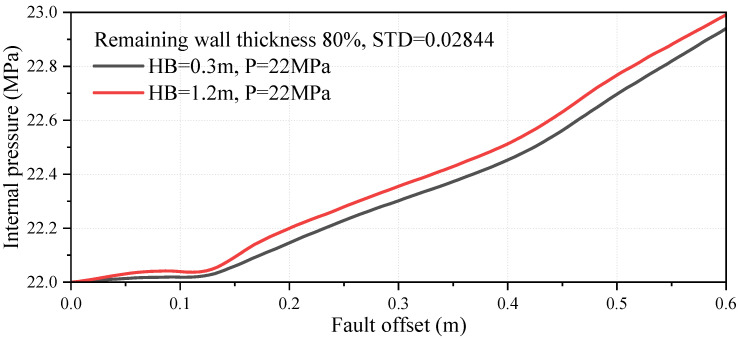
Evolution of internal pressure with fault offset under the fluid cavity approach.

To accurately capture the dynamic behavior of the internal pressurized medium and its complex interaction with the pipe wall, this study employs the fluid cavity technique. Originally developed for high-fidelity simulations of rapidly inflating structures, such as automotive airbags, this methodology treats the internal gas not as a static surface load but as a discrete thermodynamic system. In this framework, the enclosed volume of the pipeline is defined as a fluid cavity where the instantaneous pressure is governed by the state of the gas medium and its response to volume changes. This approach allows for a realistic representation of the “pneumatic support” provided by the compressed gas, particularly during the onset of local buckling or cross-sectional ovalization, where the dynamic evolution of internal pressure exerts a decisive influence on the pipeline’s post-buckling stability and failure mode transition. In contrast, a constant-pressure boundary condition enforces prescribed pressure, irrespective of deformation-driven cavity volume changes. As shown in Figure 8, the internal pressure predicted by the fluid cavity approach evolves with fault offset and increases from the initial 22 MPa to approximately 23 MPa at 0.6 m offset, with a slightly larger pressure rise observed for the deeper burial case (*HB* = 1.2 m) than for the shallower one (*HB* = 0.3 m). This indicates that the pneumatic support is not fixed during post-buckling deformation and can be affected by the pipe–soil constraint, which cannot be captured by a constant-pressure assumption.

An initial cavity pressure, P, was prescribed directly via initial conditions, while the ambient pressure was set to 0.1 MPa. This configuration established the target internal pressure state at the initiation of the analysis. The cavity fluid was modeled as an ideal gas representing Nitrogen ($N_{2}$), a standard non-reactive pressurizing medium, with a molecular weight of 0.028 kg/mol. Its molar heat capacity was characterized using a standard empirical polynomial formulation. The corresponding parameter values (listed in Table 3) were strictly adopted from the standard thermodynamic gas properties provided in the Abaqus Analysis User’s Manual [32].

To enhance the stability of the initial equilibrium state and mitigate transient oscillations during the initial increment, an initial stress field was applied to the pipe wall shell elements based on the calculated values. The longitudinal stress ($\sigma_{l}$) and hoop stress ($\sigma_{h}$) were simplified using thin-walled cylinder theory:

(5) $$\sigma_{l}=\frac{PD_{0}}{2t}$$

(6) $$\sigma_{r}=\frac{PD_{0}}{4t}$$

where *P*, *D*_0_, and *t* represent the internal pressure, outer diameter, and wall thickness of the pipeline, respectively.

### 3.4. Loading Protocol and Solution Strategy

The numerical simulations were performed using the Abaqus/Explicit dynamic solver. Given that the fault-crossing process involves severe geometric nonlinearity and complex contact state evolution, a quasi-static analysis strategy was adopted. Mass scaling techniques were strictly employed to maintain the ratio of kinetic energy to internal energy below 5%, thereby eliminating inertial effects.

The entire physical process was rigorously divided into two sequential stages: geostatic stress equilibrium with pressurization, followed by the reverse faulting movement. In the first analysis step, traditional uniform pressure loading was discarded in favor of a surface-based fluid cavity technique to inflate the pipeline. This approach treats internal pressure not as a constant boundary condition but as a dynamic variable governed by Boyle’s law. In the second analysis step, the fluid cavity remained closed, and the moving soil block (i.e., hanging wall) was driven upward along a $61^{{}^{\circ}}$ dip angle. This angle is representative of typical reverse faulting hazards. To capture critical failure characteristics within limited computational resources, the maximum fault displacement was capped at 0.6 m. This magnitude is sufficient to encompass the failure paths of local buckling or wall rupture for most scenarios, providing distinct failure modes and establishing a unified physical baseline for subsequent statistical analysis.

### 3.5. Parameter Space and Design of Simulation

To deconstruct the coupling mechanism between corrosion stochasticity and operating conditions within a multidimensional parameter space, a Monte Carlo simulation matrix comprising 3600 independent cases was constructed. The design logic does not seek an exhaustive enumeration of all variables but rather employs an organic combination of deterministic macroscopic parameters and stochastic microscopic morphology parameters to isolate the contributions of different influencing factors. Regarding deterministic dimensions: overburden thickness was fixed at 1.8 m, while burial depth was set at two levels (0.3 m and 1.2 m) to examine the nonlinear influence of soil confinement. Operating internal pressure was selected at three levels (0 MPa, 10 MPa, and 22 MPa), corresponding to 0%, 34%, and 76% of the critical yield pressure (28.77 MPa), covering the complete service cycle from maintenance shutdown to high-pressure transmission.

In the stochastic dimension, the remaining wall thickness ratios (90%, 80%, 70%) were introduced to quantify the degradation gradient of global pipeline stiffness. According to recent ILI data analytics [33], the vast majority of active and unmitigated corrosion defects in aging pipelines exhibit a relative depth between 10% and 30%. Consequently, the selected levels physically represent the ‘mild’, ‘moderate’, and ‘severe’ pre-repair metal-loss states, with the 70% threshold frequently serving as a critical operational boundary for mandatory repair or pressure derating in pipeline integrity management.

**Figure 9:**
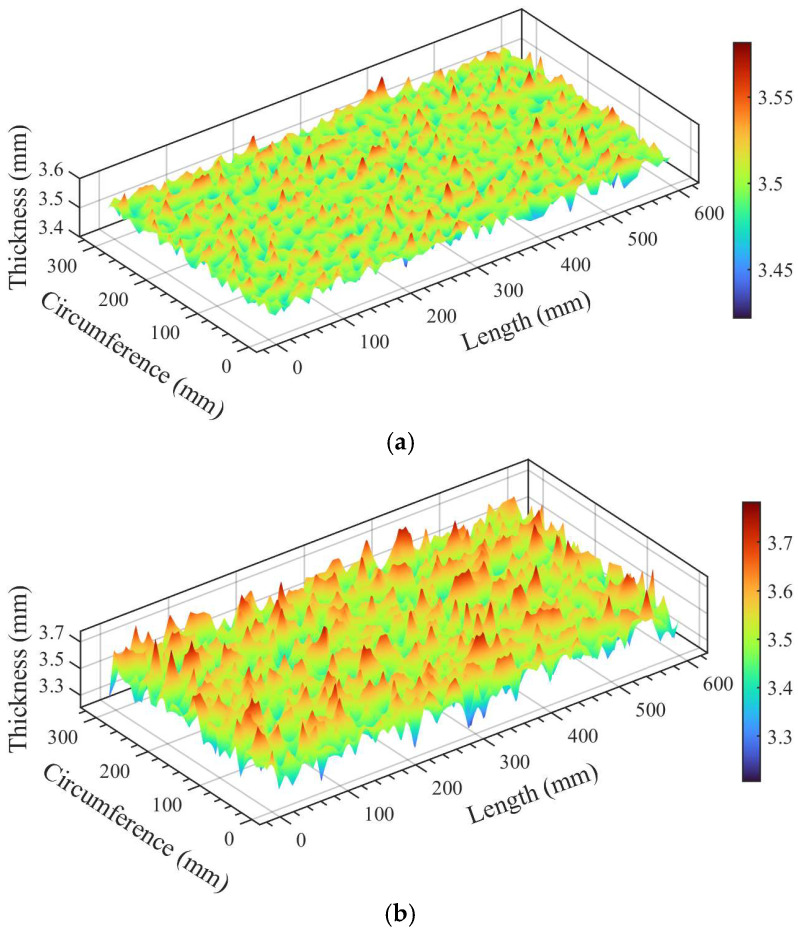
Morphological comparison of corroded pipes: (**a**) low-STD sample representing uniform corrosion, and (**b**) high-STD sample representing pitting corrosion under the same average remaining wall thickness.

Furthermore, to capture the spatial heterogeneity consistently observed in field inspections, two distinct morphology modes—low roughness (STD ≈ 0.028) and high roughness (STD ≈ 0.107)—were defined. Specifically, as illustrated in Figure 9, these values correspond to the 5% and 85% levels of the roughness distribution in the experimental dataset, strictly anchoring the parametric choices to the physical realities of relatively uniform corrosion and severe localized pitting, respectively. Recent reviews on data-driven pitting models and time-variant reliability [34,35] indicate that natural corrosion inevitably shifts from uniform wall thinning to highly localized pitting clusters over time. This high spatial variance drastically alters local stress concentration and structural degradation paths. By encapsulating this statistical transition from uniform degradation (low STD) to severe spatial pitting (high STD), the subsequent reliability assessment ensures that the simulated probabilistic outputs are methodologically rigorous and strictly anchored to field vulnerability.

Based on this parameter logic, 36 distinct operating combinations constituted the core framework of the experiment (2 HP×3 P×3 WT×2 STD). For each specific combination, the algorithm generated 100 random corrosion field samples with independent spatial distribution characteristics to ensure sufficient convergence of statistical laws. It is worth noting that due to the intrinsic randomness of field generation, the actual roughness standard deviation of these 100 samples exhibits minor fluctuations around the target value, reflecting the realistic physical volatility of natural corrosion processes.

During the post-processing phase, key physical indicators characterizing the structural limit states were systematically extracted from the complete time-history response of each stochastic sample. Two critical performance thresholds were defined to quantify the pipeline’s functional and structural integrity. First, the initiation of serviceability loss was identified by the critical fault displacement ($H_{1}$) and the corresponding instantaneous cavity pressure ($P_{1}$) at the onset of local buckling. Second, the ultimate structural limit state was determined by the displacement ($H_{2}$) and internal pressure ($P_{2}$) at the precise moment of wall rupture. Rather than adopting a constant empirical strain limit, this rupture point was explicitly governed by a stress-triaxiality-dependent damage initiation criterion to accurately capture the material ductility exhaustion under multiaxial loading. Within this framework, the critical equivalent plastic strain at fracture (${\bar{\varepsilon }}_{f}$) is defined as a decaying function of the stress triaxiality (T). Consequently, the macroscopic ultimate rupture displacement ($H_{2}$) is recorded precisely when the microscopic accumulated damage variable reaches unity ($\int d{\bar{\varepsilon }}_{p}/{\bar{\varepsilon }}_{f}(T)=1$) at the critical corrosion pit. For high-toughness samples that remained intact throughout the 0.6 m loading sequence, the corresponding datasets were treated as “right-censored” to ensure statistical rigor in the subsequent reliability analysis. Furthermore, to capture the morphological evolution of the failure modes, the topological features of the post-buckling geometry, including the maximum ovalization coefficients and the axial spacing between the primary folding kinks, were quantitatively evaluated.

## 4. Validation of Simulation Method

### 4.1. Reference Experiment Description

To validate the accuracy of the finite element modeling approach proposed in this study, a comparative analysis was conducted using the experimental and numerical results reported by Jalali et al. [36]. The cited study investigated the mechanical response of an API 5L Grade B pipeline subjected to reverse faulting. Given the similarity between their model configuration and the one presented herein, the comparative results offer a high degree of reliability. The specific case of a 4-inch diameter pipeline was selected for this validation.

In the finite element model, the soil domain represents dense sand, and the Mohr–Coulomb model was employed to characterize its elastoplastic behavior. The pipeline component employs the von Mises plasticity model with isotropic hardening. The specific constitutive model parameters for both the soil and the steel material are detailed in Table 4. Furthermore, the model accounts for the pressure-dependency of the soil’s elastic modulus, $E_{s0}$, as influenced by the hydrostatic pressure, p [36]. This relationship is expressed in Equation (7):

(7) $$E_{s0}=8356{\left(\frac{p}{100}\right)}^{0.3}$$

where p and $E_{s0}$ are expressed in kPa. In the finite element simulation, the dependency of the elastic modulus on hydrostatic pressure was implemented using the VUSDFLD user subroutine (Abaqus/Explicit). Since the reference experiment did not consider the effects of internal pressure on the pipeline response, the fluid cavity technique was not utilized in this specific validation model.

### 4.2. Comparative Analysis and Phenomenological Validation

**Figure 10:**
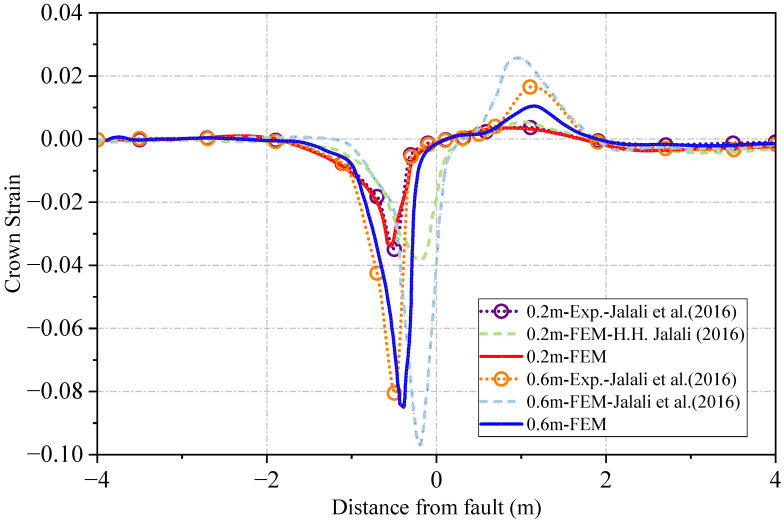
Distribution of longitudinal strain along the pipeline crown [36].

**Figure 11:**
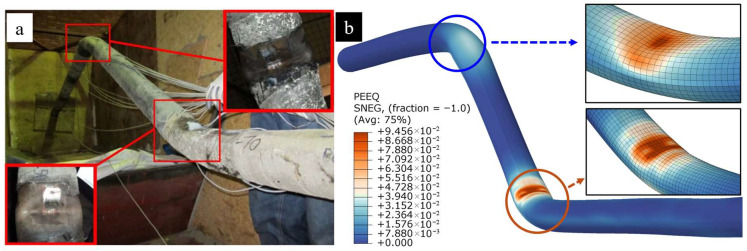
Comparison of global pipeline deformation and local buckling modes with experimental results: (**a**) experimental results [36]; (**b**) simulation results.

Comparisons between the finite element model (FEM) established in this study and the experimental and numerical results reported by Jalali et al. [36] are presented in Figure 10 and Figure 11. Figure 10 illustrates the distribution of longitudinal strain along the pipe crown. It is evident that the simulation results from the present study exhibit a high degree of agreement with Jalali’s experimental data. Furthermore, attributable to the implementation of a more refined pipe–soil contact model and a higher-fidelity mesh, the current analysis demonstrates superior accuracy compared to the numerical simulations originally performed by Jalali et al. [36].

Additionally, in the physical experiment, the soil displacement was driven by an external frame, consequently, simplified boundary conditions at the fault interface could not accurately represent the actual loading history of the soil box. To address this discrepancy, the numerical model in this validation phase released constraints across a specific width at the transition zone between the loading and fixed regions. This adjustment resulted in a closer approximation to the experimental observations. It should be noted, however, that this boundary artifact is not a governing parameter for the fundamental mechanical response of the pipeline. Therefore, the subsequent parametric analysis adopts the idealized reverse faulting boundary conditions as described in Section 3.1.

Figure 11 compares the global pipeline deformation and local buckling configurations with the experimental counterparts. The results indicate that the present FEM accurately captures the deformation characteristics under reverse faulting. Moreover, both the location and morphology of the local buckling show high consistency with the experimental findings.

## 5. Results and Discussion

This section analyzes the mechanical response of pipelines subjected to the coupled effects of random corrosion and reverse faulting, based on the Monte Carlo simulation results. First, the statistical distribution characteristics of the critical buckling displacement are discussed, quantifying the sensitivity of limit states to internal pressure, burial depth, and corrosion parameters. Subsequently, the failure mechanisms governed by strain localization and geometric distortion are elucidated using indices such as the deformed pipe length and cross-sectional ovalization. Finally, by integrating seismic fragility curves with a comprehensive failure mode map, the critical conditions precipitating the transition from a buckling-dominated to a rupture-dominated failure mode are determined.

### 5.1. Statistical Distribution and Sensitivity of Critical Displacement

**Figure 12:**
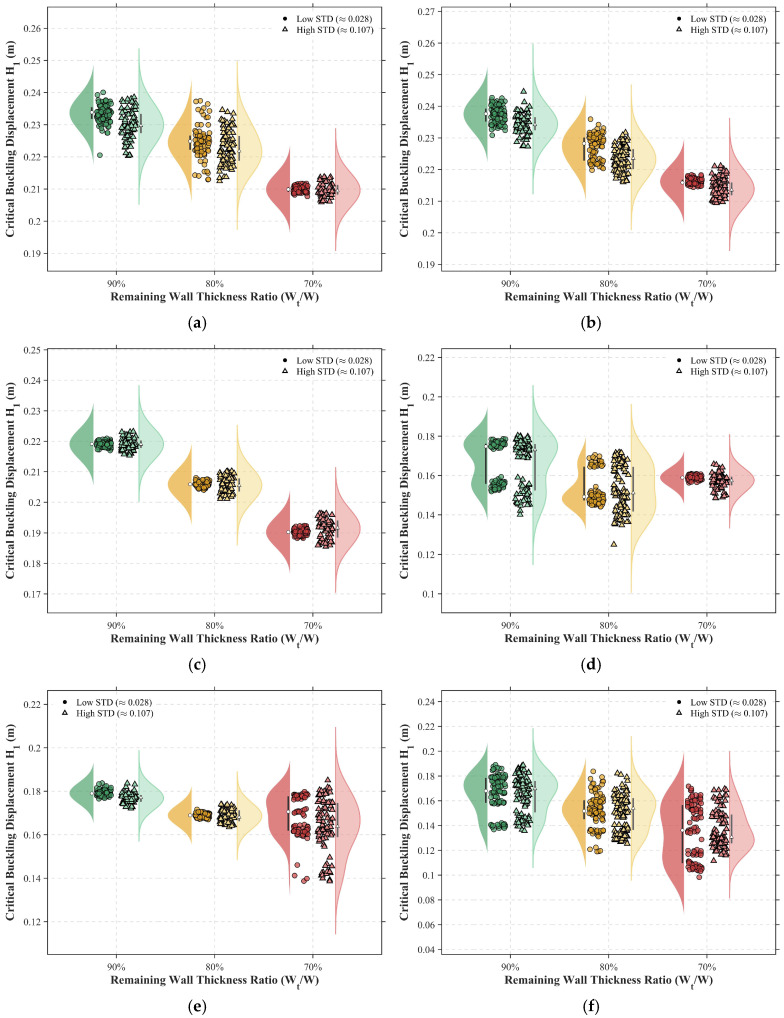
Distribution characteristics of critical buckling displacement (*H*_1_) for corroded pipelines under various burial depth and internal pressure combinations. (**a**) *HB* = 0.3 m, *P* = 0 MPa; (**b**) *HB* = 0.3 m, *P* = 10 MPa; (**c**) *HB* = 0.3 m, *P* = 22 MPa; (**d**) *HB* = 1.2 m, *P* = 0 MPa; (**e**) *HB* = 1.2 m, *P* = 10 MPa; (**f**) *HB* = 1.2 m, *P* = 22 MPa.

The statistical evolution of the critical buckling displacement, $H_{1}$, as visualized through the “raincloud plots” in Figure 12, underscores a fundamental shift in structural reliability driven by the synergistic effects of corrosion severity, surface morphology, and soil confinement.

As an initial observation, a systematic reduction in the remaining wall thickness (WT) from 90% to 70% precipitates a clear migration of the $H_{1}$ distribution toward lower thresholds, reflecting the substantial impairment of the pipeline’s global buckling resistance caused by wall thinning. For instance, under shallow burial (*HB* = 0.3 m) and zero-pressure conditions (*P* = 0 MPa), the distribution center descends from approximately 0.23 m to 0.21 m as the wall thickness reduces.

Crucially, this deterministic degradation is compounded by the stochasticity of the corrosion surface, where the standard deviation (STD) of the roughness governs the dispersion of the structural response. A comparative analysis of the probability density profiles reveals that high-roughness surfaces (High STD) consistently yield broader distribution ranges than their low-roughness (Low STD) counterparts. This effect is particularly pronounced in the *P* = 10 MPa scenario (Figure 12b), where the Low STD group exhibits a concentrated response with a narrow interquartile range (IQR), whereas the High STD group displays a distinct “tailing” effect characterized by skewness toward lower displacement values. Such statistical behavior suggests that localized pitting, inherent in non-uniform corrosion morphologies, introduces profound uncertainty into the structural limit state, causing specific pipeline segments to succumb to instability at fault displacements significantly below predicted averages.

Beyond the influence of corrosion, burial depth exerts a definitive suppressive effect on the pipeline’s deformation capacity. A comparison between shallow (*HB* = 0.3 m) and deep burial (*HB* = 1.2 m) configurations (Figure 12a–c vs. Figure 12d–f) indicates that the critical buckling displacement for deep-buried pipelines is consistently lower than that of their shallow-buried counterparts. Under identical internal pressure (*P* = 10 MPa) and wall thickness (WT 80%), the mean $H_{1}$ drops from 0.22 m in shallow soil to merely 0.17 m in deep soil. This disparity is mechanistically attributed to the intense lateral and vertical constraints imposed by the dense surrounding soil, which restrict the pipeline’s capacity for global bending. Consequently, the fault-induced deformation is forced to localize within a significantly narrowed span, thereby accelerating the onset of local wall instability and reducing the overall structural ductility.

**Figure 13:**
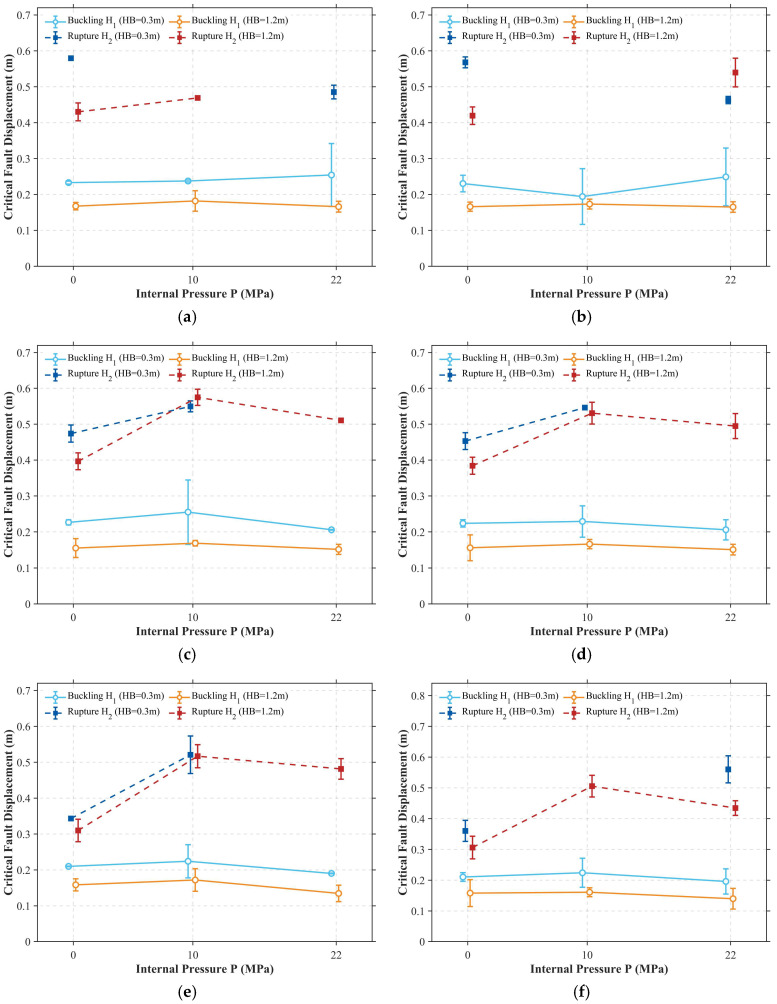
Influence of internal pressure on the critical buckling and rupture displacements of pipelines with varying degrees of corrosion and surface roughness. (**a**) WT = 90%, STD ≈ 0.028; (**b**) WT = 90%, STD ≈ 0.107; (**c**) WT = 80%, STD ≈ 0.028; (**d**) WT = 80%, STD ≈ 0.107; (**e**) WT = 70%, STD ≈ 0.028; (**f**) WT = 70%, STD ≈ 0.107.

Figure 13 elucidates the influence of internal pressure on the critical buckling displacement ($H_{1}$) and the ultimate rupture displacement ($H_{2}$) across varying corrosion intensities. As established in Section 3.5, $H_{2}$ does not represent a fixed geometric limit, but rather the macroscopic structural fault displacement at which the local accumulated damage (governed by the stress-triaxiality-dependent fracture criterion) reaches unity at the critical corrosion pit, signifying wall tearing. Each data point represents the ensemble average derived from 100 stochastic samples, with absent $H_{2}$ values indicating scenarios where no rupture occurred within the 0.6 m loading limit. A striking divergence is observed in the governing mechanisms through which internal pressure modulates these two limit states. For deep-buried pipelines (solid orange lines), $H_{1}$ remains relatively insensitive to pressure fluctuations. In contrast, shallow-buried pipelines (solid light blue lines) exhibit a significant reduction in $H_{2}$ under high-pressure conditions, with the threshold dropping by as much as 0.1 m when the remaining wall thickness is 70%. This drastic reduction in $H_{2}$ physically manifests the ductility exhaustion mechanism: while internal pressure provides geometric stiffening against ovalization, the superimposed high hoop tension drastically elevates the local stress triaxiality at deep corrosion pits. Consequently, the critical material fracture strain is degraded, accelerating damage accumulation and forcing the macroscopic structural rupture ($H_{2}$) to be triggered at a much earlier stage of fault deformation.

The regulatory effect of internal pressure is even more profound when considering the critical rupture displacement, $H_{2}$. The rupture thresholds (dark dashed lines) generally follow a non-monotonic “increase-then-decrease” trajectory as pressure rises—a pattern most acute in high-corrosion, deep-burial scenarios. This competitive mechanism is best exemplified in Figure 13e (*WT* = 70%, Low STD), where increasing the pressure from 10 MPa to 22 MPa triggers a precipitous drop in the rupture displacement for deep-buried pipelines, falling from 0.52 m to approximately 0.12 m. Mechanistically, this collapse of the limit state occurs because the immense hoop tensile strain induced by high pressure superimposes onto the localized axial bending strain. Under such multi-axial loading, the pipe wall material rapidly exhausts its plastic ductility reserve. Consequently, $H_{2}$ converges toward $H_{1}$, implying a transition to a brittle-like failure mode where the pipeline ruptures almost instantaneously following the onset of buckling.

Furthermore, a comparison between Low STD (Figure 13e) and High STD (Figure 13f) morphologies reveals that the influence of surface roughness on H_2_ becomes more pronounced under high-pressure conditions. At 22 MPa, the H_2_ values for the High STD group are approximately 0.55 m for shallow-buried pipelines and 0.40 m for deep-buried pipelines, whereas the Low STD group exhibits no rupture failure under high pressure for shallow burial conditions, with deep-buried pipelines reaching approximately 0.48 m. This discrepancy indicates that local pits introduced by high surface roughness significantly undermine the geometric stiffening benefit provided by internal pressure. Under low-roughness conditions, relatively uniform wall thinning allows the pipeline to dissipate energy through global bending deformation under high pressure, thereby avoiding rupture failure in shallow burial scenarios. However, deep pits in high roughness morphologies act as stress concentration sources, inducing premature localization of local strain under the coupled action of high hoop stress and fault bending strain, leading to rupture even in shallow burial conditions. This implies that for high-roughness aged pipelines, safety assessments based on deterministic uniform corrosion assumptions may severely underestimate the rupture risk under high-pressure operation, especially under complex operating condition combinations of shallow burial and high internal pressure.

### 5.2. Failure Mechanism Analysis

#### 5.2.1. Mechanistic Interpretation of Failure Mode Transition

To mechanistically interpret the observed transition from ductile buckling to rupture-dominated failure, the interplay between Brazier ovalization and multiaxial ductility exhaustion must be quantitatively formulated.

(1) Energy-based interpretation of geometric stiffening

Under severe bending induced by reverse faulting, the longitudinal compressive stresses generate a distributed radial force that drives cross-sectional flattening, classically known as the Brazier effect [37]. To quantitatively interpret the geometric stiffening provided by internal pressure, this study adopts the energy-based framework originally established by Brazier. Based on the minimum potential energy principle, the theoretical cross-sectional ovalization ζ under an internal pressure P can be analytically approximated as:

(8) $$\zeta \propto \frac{EtR\kappa^{2}}{\frac{Et^{3}}{12(1-\nu^{2})R^{2}}+P\cdot R}$$

This energy-based formulation mathematically reveals the dual mechanism. The numerator ($EtR\kappa^{2}$) represents the flattening driving force induced by faulting. Crucially, the internal pressure acts directly as a pneumatic restoring force (P⋅R) in the denominator. At moderate pressures (e.g., 10 MPa), this restoring force effectively counteracts the flattening, thereby delaying geometric bifurcation.

It should be noted that while the classic Brazier formulation provides the fundamental theoretical mechanism, it is derived under the assumptions of pure elasticity and pristine geometry. Therefore, the actual post-buckling cross-sectional distortion extracted from the nonlinear FEA is rigorously quantified using the standard geometric ovality coefficient (Equation (9)).

(2) Stress-path evolution and ductility exhaustion

**Figure 14:**
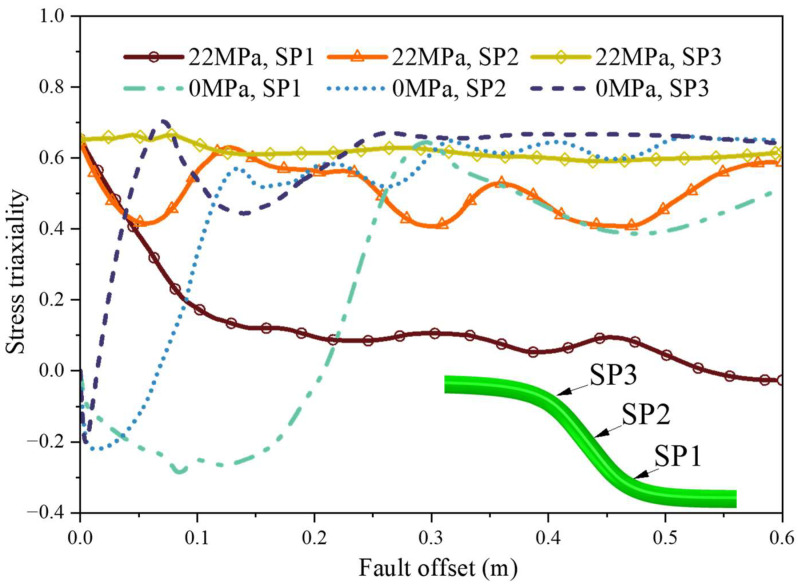
Effect of internal pressure on the evolution of stress triaxiality at local critical points.

To rigorously quantify the failure mode transition, Figure 14 illustrates the evolution paths of stress triaxiality at critical deformation regions against the fault offset. Under unpressurized conditions, the stress triaxiality in the early stage of deformation remains predominantly negative; this compression-dominated multiaxial stress state fundamentally drives the occurrence of cross-sectional ovalization and macroscopic local buckling. Conversely, under a high internal pressure of 22 MPa, the initial stress triaxiality at all monitored points is elevated to approximately 0.65 due to the severe hoop tension. Particularly, in tension-dominated regions such as SP3, tension is sustained at a high level above 0.6 throughout the entire deformation process. In conjunction with the ductile damage criterion adopted in this study, this continuous high-triaxiality stress path inherently leads to an exponential decay in the critical fracture strain of the material. This mechanistically demonstrates how the high-pressure environment accelerates ductility exhaustion, forcing the pipeline to undergo premature tensile rupture before overall global instability can fully develop, as the accumulating plastic strain rapidly reaches the degraded fracture tolerance.

The observed transition from ductile buckling to premature rupture under high internal pressure aligns with and fundamentally expands upon recent investigations into pipeline–fault interactions. For instance, recent numerical assessments by Qiu et al. [5] and related faulting studies [6] noted the dual effect of internal pressure, wherein moderate pressure enhances cross-sectional stability but excessive pressure exacerbates circumferential tensile stress. The energy-based Brazier formulation and stress triaxiality analysis quantitatively substantiate these prior phenomenological observations, revealing that the superposition of hoop tension and localized bending strain fundamentally degrades the material’s multiaxial fracture tolerance.

#### 5.2.2. Strain Localization and Geometric Distortion

**Figure 15:**
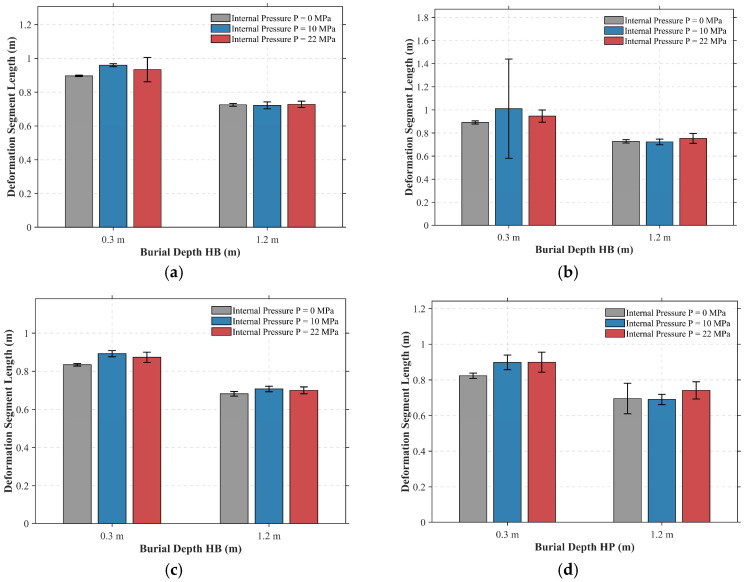

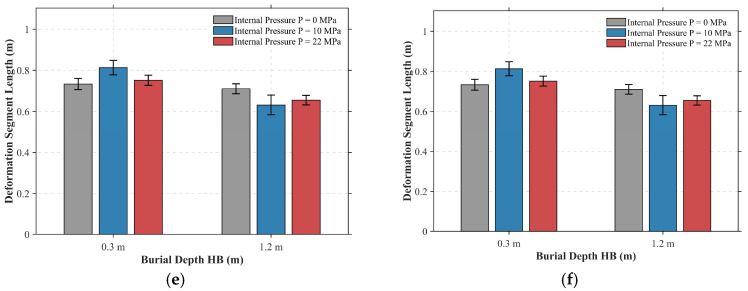
Influence of burial depth and internal pressure on the deformation segment length of pipelines with varying degrees of corrosion. (**a**) *WT* = 90%, STD ≈ 0.028; (**b**) *WT* = 90%, STD ≈ 0.107; (**c**) *WT* = 80%, STD ≈ 0.028; (**d**) *WT* = 80%, STD ≈ 0.107; (**e**) *WT* = 70%, STD ≈ 0.028; (**f**) *WT* = 70%, STD ≈ 0.107.

Building upon the macroscopic laws governing critical displacement discussed above, this section focuses on analyzing the phenomenon of strain localization induced by burial depth and the process of cross-sectional geometric distortion triggered by corrosion defects. Figure 15 presents the statistical results for the plastic deformation segment length of the pipeline under various operating conditions. Observing the consistent trends in Figure 15a–d, it is evident that under shallow burial conditions (*HB* = 0.3 m, left bar group in each subplot), the deformation segment length is generally larger. Taking Figure 15a (*WT* = 90%, Low STD) as an example, the mean deformation segment length stabilizes between 0.90 m and 0.96 m across different internal pressures. This indicates that the weaker overburden soil pressure allows the pipeline to undergo relatively gentle beam-like bending over a longer span, thereby dissipating the strain energy input from the fault movement over a broader region.

However, once the deep burial condition is introduced (*HB* = 1.2 m, right bar group in each subplot), the deformation segment length decreases significantly. In the corresponding case of Figure 15a, the length shortens to approximately 0.72 m, representing a reduction of about 25%. Similarly, in Figure 15c (*WT* = 80%), this value drops from approximately 0.85 m (shallow) to near 0.70 m (deep). This demonstrates that strong soil confinement restricts the axial and lateral sliding of the pipeline. Consequently, the same magnitude of fault displacement must be accommodated by a shorter pipe segment, leading to a sharp increase in local curvature and axial compressive strain, which in turn induces severe strain localization.

It is noteworthy that internal pressure exhibits a significant nonlinear regulatory effect on the deformation mode, particularly under conditions of shallow burial and severe corrosion. As observed in the shallow burial groups (*HB* = 0.3 m) of Figure 15f, under lower internal pressures (0 MPa and 10 MPa), the mean deformation segment length remains at a low level of 0.7 m to 0.8 m. This indicates that thinner walls tend to undergo more concentrated buckling. However, when the internal pressure increases to 22 MPa, the mean deformation length exhibits an abrupt jump to 2.1 m (Figure 15f), accompanied by dispersion. This anomalous phenomenon suggests that the strong “geometric stiffening effect” generated by high internal pressure counteracts, to a certain extent, the structural softening caused by wall thinning, attempting to maintain the pipeline in a state of long-wave global bending. However, due to the excessive weakness of the pipe wall (*WT* = 70%), this pneumatic support is highly unstable. Consequently, the structure resides at a critical bifurcation point between “global bending” and “local instability,” manifested as violent fluctuations in the deformation range.

**Figure 16:**
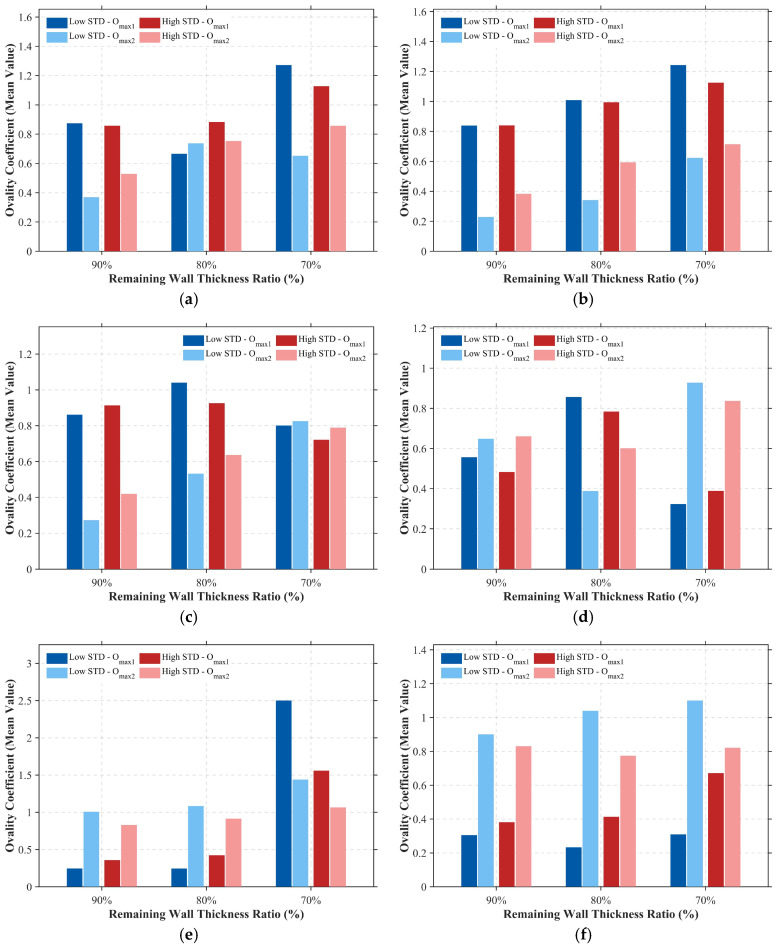
Influence of corrosion parameters on the maximum cross-sectional ovality coefficient at the dual kink locations under various burial depth and internal pressure conditions. (**a**) Burial depth *HB* = 0.3 m, Internal pressure *P* = 0 MPa; (**b**) Burial depth *HB* = 0.3 m, Internal pressure *P* = 10 MPa; (**c**) Burial depth *HB* = 0.3 m, Internal pressure *P* = 22 MPa; (**d**) Burial depth *HB* = 1.2 m, Internal pressure *P* = 0 MPa; (**e**) Burial depth *HB* = 1.2 m, Internal pressure *P* = 10 MPa; (**f**) Burial depth *HB* = 1.2 m, Internal pressure *P* = 22 MPa.

The impairment of pipeline load-bearing capacity by corrosion defects is manifested not only in the strength degradation caused by wall thinning but, more critically, in the induced cross-sectional geometric distortion. Figure 16 quantifies this distortion process using the maximum Ovality Coefficient at the dual kink locations of the pipeline; the calculation formula is presented in Equation (9). As the remaining wall thickness decreases from 90% to 70%, the ovality coefficient exhibits a nonlinear increase across all operating conditions. Particularly under deep burial and severe corrosion scenarios (Figure 16e,f), the ovality escalates sharply, indicating that the pipe wall has effectively lost its capacity to resist radial collapse.

(9) $$f=\frac{D_{max}-D_{min}}{D_{max}+D_{min}}\times 2$$

where $D_{\max}$ and $D_{\min}$ represent the maximum and minimum outer diameters of the same cross-section after deformation, respectively.

A comparison of data across different roughness groups reveals that, in most operating conditions, the height of the dark blue columns (representing Low STD, $O_{\max1}$) exceeds that of the dark red columns (representing High STD, *O*_max1_). Taking the data for *WT* = 70% in Figure 16e (*HB* = 1.2 m, *P* = 10 MPa) as an example: the mean ovality for the Low STD group reaches as high as 2.5, indicating extremely severe cross-sectional flattening and folding. In contrast, the ovality for the High STD group is only approximately 1.5.

Low roughness corresponds to relatively uniform wall thinning. This geometric “regularity” allows the pipeline cross-section, when subjected to bending compression, to undergo a coordinated global flattening mode. Consequently, it can develop significant ovalization without immediate local rupture. Conversely, the dense, deep pitting associated with high roughness (High STD) disrupts the geometric continuity of the cross-section, causing severe stress concentration. These local defects often induce wall tearing or local buckling before the cross-section can fully flatten, thereby limiting the further development of ovality.

Comparing the medium pressure case (Figure 16e, 10 MPa) with the high-pressure case (Figure 16f, 22 MPa), it is evident that when internal pressure rises to 22 MPa, the overall ovality level is significantly suppressed (dropping from 2.5 to below 1.2). This reconfirms that higher operating pressure maintains the circularity of the pipeline cross-section and restricts radial collapse; however, this comes at the cost of converting more energy into tensile strain within the pipe wall, thereby increasing the risk of rupture.

In summary, the collective evidence from Figure 15 and Figure 16 elucidates a complex hierarchy of failure mechanisms governed by both environmental and morphological factors. The intense confinement characteristic of deep burial environments predominantly drives macroscopic strain localization, whereas the specific nature of the corrosion significantly modulates the response at the local level. Specifically, uniform corrosion, associated with low surface roughness, facilitates severe and coordinated geometric distortion of the cross-section. In contrast, pitting corrosion with high roughness tends to trigger early-stage local failure by disrupting geometric continuity and inducing premature stress concentrations. Recognizing this fundamental differentiation in failure modes is essential for accurately predicting the limit behavior of pipelines across the diverse spectrum of corrosion morphologies encountered in the field.

### 5.3. Fragility Assessment and Failure Mode Evolution

This section aims to translate the aforementioned mechanical response into probabilistic risk indicators, quantifying the failure probability and ultimate failure modes of pipelines under reverse faulting. By constructing seismic fragility curves for the rupture limit state ($H_{2}$) and integrating a panoramic statistical view of failure modes, the comprehensive seismic risk under different combinations of corrosion severity and operating conditions is systematically assessed. This allows for the definition of critical conditions governing the transition from ductile buckling to brittle rupture.

**Figure 17:**
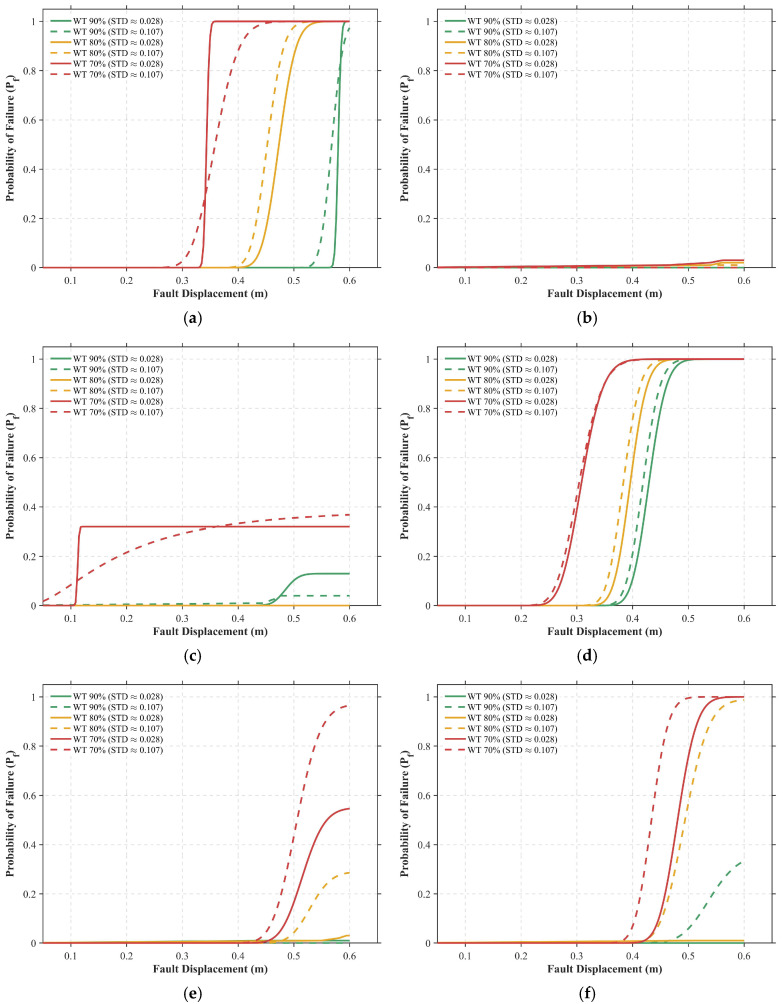
Rupture fragility curves for corroded pipelines under 0.6 m reverse faulting displacement. (**a**) Burial depth *HB* = 0.3 m, Internal pressure *P* = 0 MPa; (**b**) Burial depth *HB* = 0.3 m, Internal pressure *P* = 10 MPa; (**c**) Burial depth *HB* = 0.3 m, Internal pressure *P* = 22 MPa; (**d**) Burial depth *HB* = 1.2 m, Internal pressure *P* = 0 MPa; (**e**) Burial depth *HB* = 1.2 m, Internal pressure *P* = 10 MPa; (**f**) Burial depth *HB* = 1.2 m, Internal pressure *P* = 22 MPa.

The seismic fragility curves presented in Figure 17 quantify the exceedance probability of pipelines reaching the rupture limit state as a function of fault displacement. These probabilistic profiles underscore a significant escalation in structural vulnerability driven by the synergistic effects of wall thinning and increased surface roughness. Across all investigated conditions (Figure 17a–f), the fragility curves associated with severe corrosion (*WT* = 70%, represented in red) are consistently shifted toward the lower displacement spectrum, indicating a substantial reduction in the seismic margin. For instance, in the deep-burial, high-pressure scenario (Figure 17f), a pipeline with 90% remaining wall thickness maintains a nearly zero failure probability at 0.45 m displacement; however, reducing the thickness to 70% causes the failure probability to escalate to 100% at the same loading level. This trend is further exacerbated by high surface roughness (High STD), which introduces a “low-value extension” in the fragility response. As demonstrated in Figure 17d, the low-roughness *WT* = 80% sample exhibits a failure probability ($P_{f}$) of only 10% at 0.35 m displacement, whereas its high-roughness counterpart reaches 80%, reinforcing the conclusion that neglecting the stochastic nature of corrosion leads to a critical underestimation of rupture risk.

The spatial dispersion of corrosion morphology further modulates the estimated failure probability, acting as a primary driver of the statistical “tailing” effect. In most scenarios, high-roughness profiles (dashed lines) are positioned to the left of their low-roughness counterparts (solid lines), indicating an earlier onset of rupture. This phenomenon is particularly striking in the medium-pressure, deep-burial case (Figure 17e) for *WT* = 80%: while the low-roughness model predicts negligible risk throughout the 0.6 m loading range, the high-roughness model reveals a $P_{f}$ exceeding 30% by the end of the sequence. Mechanistically, these results suggest that for an identical average wall thickness, localized pitting associated with high roughness creates high-sensitivity initiation sites for stress concentration. These local defects trigger premature rupture at displacements far below the deterministic average, suggesting that relying solely on low-variance models may overlook significant high-consequence failure risks in aged infrastructure.

Furthermore, internal pressure exerts a complex, non-monotonic influence on pipeline fragility, dictated by the competition between geometric stabilization and tensile strain accumulation. Fragility curves for zero-pressure conditions (*P* = 0 MPa, Figure 17a,d) exhibit relatively steep slopes, reflecting a high sensitivity to rupture induced by severe sectional folding. In contrast, the medium-pressure regime (*P* = 10 MPa, Figure 17b,e) displays the most gradual curve morphologies, with rupture events becoming almost nonexistent under shallow burial (Figure 17b). This implies that moderate internal pressure provides an optimal level of geometric stiffness that effectively delays the concentration of local strains. However, this protective effect vanishes under high-pressure conditions (*P* = 22 MPa, Figure 17c,f), where the fragility profiles deteriorate significantly. In deep-burial scenarios (Figure 17f), these curves shift even further toward the lower displacement range than the zero-pressure baseline. This shift indicates that the superposition of high hoop stress and axial bending strain compels the material to reach its fracture limit at minimal displacements, thereby defining the critical operational boundary for aged, fault-crossing pipelines.

**Figure 18:**
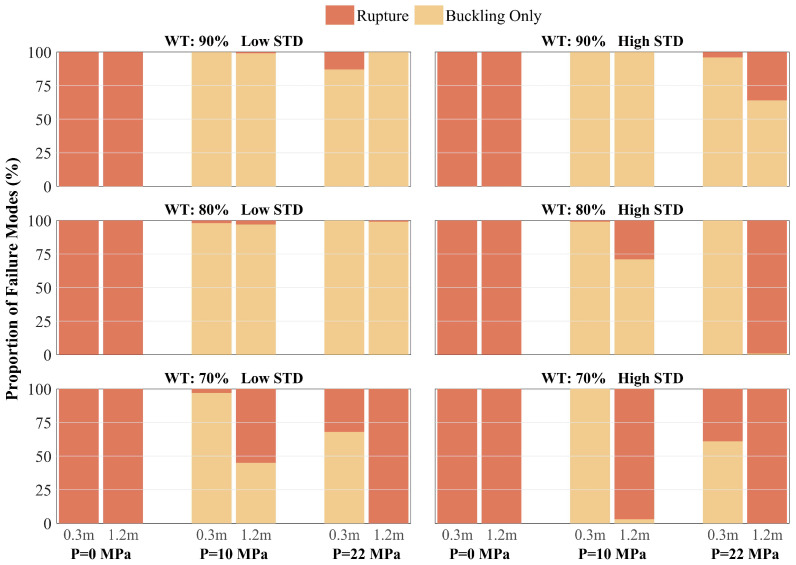
Proportional distribution of pipeline failure modes under 0.6 m fault displacement across various combinations of corrosion and operating parameters.

To elucidate the statistical transition of the ultimate structural state, Figure 18 presents a panoramic distribution of failure modes—specifically differentiating “Buckling Only” (yellow) from “Rupture” (red)—across the investigated parameter space. This faceted stacked bar chart reveals that under zero-pressure conditions (*P* = 0 MPa), the pipeline exhibits a near-total susceptibility to rupture, with the failure proportion approaching 100% across all wall thicknesses and roughness levels. Mechanistically, the absence of “pneumatic support” renders the pipeline cross-section vulnerable to rapid flattening and sectional collapse upon fault movement. This structural instability induces extreme local curvature and severe stress concentration at the folding kinks, which subsequently triggers localized tearing. In this unpressurized regime, the pipeline demonstrates highly brittle failure characteristics that remain largely indifferent to variations in burial depth.

A fundamental shift in the failure trajectory occurs as the internal pressure increases to a moderate level of 10 MPa. In this regime, the “Buckling Only” domain expands significantly, becoming the dominant state. Notably, under shallow burial (0.3 m) and mild corrosion (*WT* = 90%), the rupture probability drops to zero. This observation implies that moderate internal pressure effectively counteracts excessive cross-sectional distortion—the Brazier effect—allowing the pipeline to maintain superior circularity and undergo ductile beam-mode bending. While local buckling may still initiate, the structure retains substantial integrity, defining an optimal operating window for fault-crossing resilience.

However, further pressurization to 22 MPa triggers a secondary transition, wherein the rupture domain expands once again, particularly under severe corrosion and deep burial conditions. In the most critical combination (*WT* = 70%, High STD, *HB* = 1.2 m), the rupture rate reverts to 100%, driven by a mechanism that differs fundamentally from the zero-pressure scenario. In this high-pressure regime, the failure is dominated by intense hoop tensile stresses that deplete the material’s axial ductility reserve, leading to fracture. The comparative analysis across burial depths confirms that deep soil confinement consistently elevates the risk of rupture by restricting global deformation. Collectively, these results demonstrate that the synergy between intense soil constraint, geometric defects from severe corrosion, and high-pressure-induced ductility exhaustion serves as the primary driver forcing the failure mode transition from ductile buckling to catastrophic rupture.

Taken together, the synthesis of fragility assessments and failure mode distributions in Figure 17 and Figure 18 elucidates the critical governing principles for the design of fault-crossing pipelines. The findings define two distinct failure domains characterizing the structural limits: sectional collapse driven by a lack of internal support at zero pressure, and material fracture driven by ductility exhaustion under high-pressure operations. Notably, the medium-pressure regime emerges as a vital “buffer window,” where the synergy of geometric stabilization and manageable tensile strain offers the most resilient state against fault-induced deformation. However, this resilience is highly sensitive to the pipeline’s service history; for infrastructure nearing the end of its design life (WT≤70%), particularly when subjected to the intense confinement of deep burial, the risk of sudden, brittle-like rupture under high-pressure conditions is markedly amplified. Consequently, these results necessitate a shift toward risk-informed maintenance strategies that prioritize the monitoring of operating pressure and burial environments for aged pipelines, ensuring their continued integrity within complex geological corridors.

### 5.4. Limitations and Future Work

Although the proposed probabilistic framework comprehensively elucidates the coupled effects of internal pressure and stochastic corrosion morphologies, it represents a foundational step toward fully capturing the complexities of real-world fault–pipeline interactions. Several critical variables fall beyond the current parametric scope. Factors such as the diameter-to-thickness (D/t) ratio, broader ranges of corrosion severity, varied geotechnical soil conditions, alternative pipeline materials, and diverse fault dip angles can significantly govern structural performance and failure mechanisms. As highlighted by recent advanced studies in this domain [38], expanding the analytical space to encompass these multifaceted uncertainties is crucial. Furthermore, translating the current theoretical fragility results into mature, actionable pressure-corrosion thresholds for field maintenance strictly requires extensive empirical validation.

To address these limitations, future extensions of this research will focus on two primary objectives. First, the aforementioned geometric and geotechnical variables will be systematically investigated to enhance the generalizability and practical applicability of the reliability assessments. Second, ongoing work by the authors involves conducting full-scale fault-crossing experiments under coupled multiphysics conditions. These physical efforts will serve to rigorously calibrate the numerical fragility curves, ultimately transforming this foundational framework into definitive, risk-informed inspection guidelines for aging energy infrastructure.

## 6. Conclusions

This study establishes a comprehensive probabilistic framework to elucidate the nonlinear mechanical response of buried pressurized pipelines under the coupled influence of stochastic corrosion and large ground deformation. The analysis reveals that the structural integrity of these energy lifelines is governed by a complex interplay between geometric stiffening, soil confinement, and spatial heterogeneity of material degradation. The specific findings are summarized as follows:

(1) The dual role of internal pressure in modulating failure mechanisms. Internal pressure exerts a significant geometric stiffening effect during the large-deformation phase of reverse faulting, which effectively enhances the pipeline’s adaptive capacity. Mechanistically, moderate operating pressure generates hoop tensile stresses that counteract the Brazier effect, thereby suppressing cross-sectional distortion and facilitating a transition from severe localized wrinkling to a more uniform beam-mode bending. However, excessive pressurization shifts the failure regime from ductile buckling to premature tensile rupture, as the pre-tension stress accelerates the exhaustion of axial ductility at critical sections.
(2) The decisive influence of soil confinement on strain localization. Burial depth significantly alters the deformation topology through soil–structure interaction. Compared to shallow burial, deep soil imposes strong circumferential constraints that hinder the development of global bending, forcing fault-induced displacement to concentrate within a highly localized pipe segment. Consequently, this “confinement-induced localization” triggers a rapid accumulation of axial strain, leading to structural instability at significantly smaller fault displacements—a risk that is further exacerbated under the coupled action of high pressure and severe corrosion.
(3) The stochastic nature of corrosion as a driver of reliability dispersion. The spatial variability of corrosion morphology, which is captured in this study through a generative conditional diffusion model, represents a primary driver of wall strain localization. In contrast to traditional models based on average wall thinning, these results demonstrate that surface roughness significantly amplifies the dispersion of critical limit states rather than merely lowering their mean values. This finding suggests that the stochastic distribution of local pits acts as a set of high-sensitivity initiation sites for stress concentration, thereby governing the “long-tail” risk of premature rupture that is often overlooked in deterministic assessments.
(4) The necessity of a probabilistic paradigm in safety assessment. Fragility analysis indicates that deterministic safety factors fail to capture the catastrophic risks associated with high-roughness corrosion. While moderate internal pressure offers a “buffer window” by maintaining circularity, the synergy between deep burial and high-pressure operation creates a high-vulnerability regime for aged pipelines. Collectively, these findings provide a quantitative scientific basis for the risk-informed design and life-extension decision-making of energy infrastructure facing complex geological threats.

In summary, this research elucidates the fundamental mechanics governing the nonlinear interaction between stochastic degradation and large-scale ground movement; moreover, it establishes a quantitative paradigm for the risk-informed design and life-extension of energy infrastructure facing complex geological threats. By bridging morphology modeling with high-fidelity structural analysis, this work provides a transformative framework for ensuring the resilience of global lifeline systems in an era of increasing geological and environmental uncertainty.

## Figures and Tables

**Table 1:** Material parameters of the pipeline.

Pipe Parameter	Value
Density, *ρ* (kg/m^3^)	7850
Young’s modulus, *E* (GPa)	206
Poisson’s ratio, *υ*	0.3
Yield strength, *σ*_y_ (MPa)	406
Ultimate strength, *σ*_u_ (MPa)	485

**Table 2:** Material parameters of the soil.

Soil Parameter	Value
Density, *ρ* (kg/m^3^)	1790
Young’s modulus, *E* (MPa)	180
Poisson’s ratio, *υ*	0.33
Friction angle, *φ* (degree)	33.5
Cohesion stress, *c* (kPa)	250

**Table 3:** Molar heat capacity coefficients.

Coefficients	Value
Constant term *C*_0_	26.092
Linear term *C*_1_	0.008218
Quadratic term *C*_2_	−1.976 × 10^−6^
Cubic term *C*_3_	1.592 × 10^−10^
Inverse quadratic term *C*_4_	44,400

**Table 4:** Finite Element Model Parameters for Validation.

	Parameter	Value
Fault Parameters	Fault-Pipeline Crossing Angle *β* (°)	60
	Fault Displacement *δ* (m)	0.6
Soil Parameters	Soil Domain Dimensions (m × m × m)	1.7 × 2.0 × 8.5
	Density *ρ* (kg/m^3^)	1870
	Poisson’s Ratio *μ*	0.3
	Friction Angle *φ* (°)	33.5
	Dilation Angle *Ψ* (°)	3.5
	Cohesion *c* (kPa)	5
Pipeline Parameters	Material Grade	API/5L Grade B
	Yield Stress *σ_s_* (MPa)	450
	Elastic Modulus *E_st_* (GPa)	200
	Poisson’s Ratio *μ*	0.3
	Diameter *D* (mm)	101.6
	Wall Thickness *t* (mm)	3.6
	Burial Depth *h* (m)	1.2

## Data Availability

The original contributions presented in this study are included in the article. Further inquiries can be directed to the corresponding author.
